# The Role of Permission, Supervision, and Precipitating Events in Childhood Pool/Spa Submersion Incidents, United States, 2000–2017

**DOI:** 10.3390/ijerph18168776

**Published:** 2021-08-19

**Authors:** Kristina R. Anderson, William D. Ramos, James T. Schuman

**Affiliations:** 1 Department of Health & Wellness Design, School of Public Health, Indiana University-Bloomington, 1025 E. 7th Street, Bloomington, IN 47405, USA; wramos@indiana.edu; 2 Eppley Institute for Parks and Public Lands, Indiana University-Bloomington, 2805 E. 10th Street, Bloomington, IN 47408, USA; 3 Aquatics Institute, School of Public Health, Indiana University-Bloomington, 1025 E. 7th Street, Bloomington, IN 47405, USA; 4 Department of Physician Assistant Studies, School of Health & Human Sciences, Indiana University-Purdue University Indianapolis, 901 W. New York Street, Indianapolis, IN 47405, USA; jimschum@iu.edu

**Keywords:** submersion, drowning, injury prevention, supervision, children

## Abstract

Drowning is a leading cause of fatality among children in the United States, and pool/spa aquatic structures represent common locations of submersion incidents. This study employed narrative case review to understand characteristics related to permission, supervision, and precipitating events in childhood submersion incidents. Retroactive analysis of 1537 fatal and non-fatal submersion incidents among children age 13 years old and younger was conducted using the U.S. Consumer Products Safety Commission In-Depth Investigations dataset from 2000–2017. Narrative descriptions were coded according to the themes of permission, supervision, and precipitating events. In most (86%) incidents, the child did not have permitted water access, and 80% of narratives indicated the child was alone at time of incident. These attributes were significantly associated with a fatal outcome (No permission: OR 11.98, 95% CI 7.97–18.06; Alone: OR 34.93, 95% CI 19.69–61.96). The average length of inactive supervision time was 15.6 min; this duration significantly differed by non-fatal (3.2 min) and fatal (16.1 min) outcomes (*p* < 0.001). More than half of cases occurred under the supervision type of a parent (56%), followed by grandparents (14%) and childcare provider (10%). Submersion incidents with a non-parent supervisor were two times more likely to result in a fatal outcome (OR 1.87, 95% CI 1.07–3.64). The most frequently occurring precipitating events included outdoor play (46%), a social gathering (36%), and previous water play (15%). Narrative excerpts further illustrate how tragic submersion events can unfold quickly and unpredictably. Education campaigns should target all adults that supervise children and reiterate key findings in that many submersion incidents occur (1) without permitted pool use, (2) without active supervision, and (3) when a caregiver is distracted. Multiple strategies should be utilized to add layers of projection against submersion injury.

## 1. Introduction

Among children ages 1–4, drowning is the leading cause of fatal injuries in the United States (U.S.) [1], and in reports of unintentional drownings involving swimming pools across several countries, the U.S. ranks highest (18%) [2,3]. Whereas drowning is defined as “the process of experiencing respiratory impairment from submersion/immersion in liquid” and its associated outcomes are death, morbidity, and no morbidity, submersion describes all situations in which a victim’s airway is under water [4]. Across various water settings, pools and spas (i.e., home spas or hot tubs) in private and residential backyards are frequently implicated sites of unintentional drowning and submersion incidents, especially among young children [5,6,7]. In contrast to pools and spas at locations such as public pools or hotels, private and residential pools and spas are particularly noteworthy due to their proximate location to everyday home life and where the nature of the supervision differs (e.g., caretakers who are generally not trained in water safety and/or engaged in other tasks).

Much of what is known regarding drowning deaths is derived from a wide variety of datasets and/or relational databases, such as the Web-based Injury Statistics Query and Reporting System (U.S.) or the Surfguard Incident Report Database (Australia). Research efforts to classify youth drowning and submersion incidents often categorize case reports by demographic factors, such as age and gender of the victim. In the U.S., toddler/preschool years and male gender are generally known risk factors associated with drowning-related hospital admissions and deaths [8,9,10]. However, less is understood regarding behavioral factors associated with drowning incidents, particularly those related to permission, supervision, and other relevant precipitating events. Supervision, in particular, is crucial in preventing children’s permitted access to water and represents a layer of protection that “should be ever-present no matter what other layers are utilized” [11].

Understanding the role of these factors (permission, supervision, and precipitating events) in U.S. childhood drownings is an area of limited research, yet use of existing datasets can illuminate crucial epidemiological insight into submersion incidents and related outcomes. Shields, Pollack-Nelson, and Smith’s [12]’s work evaluating 244 U.S. submersion cases by supervisor (e.g., adult, child) and lapse in supervision (documented or undocumented) found that nearly 40% of cases occurred while a child was unsupervised, and another 19% occurred during a lapse in supervision. However, their research was limited to only portable, above-ground pools and did not make additional distinctions, such as supervisor gender or the child’s activity prior to the incident. Research in Australia has evaluated case report investigations at regional and national levels to better understand the role of these factors in childhood drowning incidents [13]. In one study reviewing 33 toddler death reports in the Australia state of Victoria, 85% of cases occurred while the child was in the care of one or both parents; and in 45% of cases, an interruption in supervision occurred, such as a caretaker performing a household chore or engaging in a social interaction like taking a phone call [13]. More recently, work reviewing 426 drowning cases of young children in Australia during a 15-year span (2002–2017) also found that indoor (28%) and outdoor (13%) household duties as well as talking or socializing with others (12%) were frequent cases of supervisor distraction [14]. That study also indicated that incidents frequently occurred when the child was in the company of one or more other children (40%), and 7% drowned when a grandparent was serving as a primary supervisor; during parental supervision incidents, the mother was identified as the individual primarily responsible for supervision 49% of the time, differing substantially from fathers (24%) [14]. Still, much of the research to date on the role of permission, supervision, and precipitating events in youth submersion incidents has been limited to specific settings [12], limited categorization of these variables [15], and/or non-U.S., international contexts [13,14].

Literature has indicated that supervision is the primary injury-prevention strategy among caregivers, particularly in their care for young children [16]. However, most studies seeking to understand supervision in aquatic environments have focused on limited dimensions of supervision, such as the presence or absence of supervisor continuity [16]. Through detailed review of descriptive narratives about submersion incidents occurring in pools and spas, this study identifies and categorizes multiple attributes associated with permission, supervision, and precipitating events in fatal and non-fatal submersion incidents, as reported to the U.S. Consumer Product Safety Commission (CPSC) from 2000–2017. While this dataset is characterized by sampling biases deriving from details excluded in incident narratives, to the best of our knowledge, this is the first large-scale study to apply such an epidemiological lens to youth submersion incidents based on these three attribute areas. In doing so, we sought to identify supervisory and circumstantial elements associated with youth submersion incidents to inform injury-prevention efforts.

## 2. Materials and Methods

### 2.1. Sample

The Consumer Product Safety Commission (CPSC) In-Depth Investigations (INDP) dataset represents the source of information for the study’s retrospective analysis. As a convenience sample, the INDP dataset includes cases identified through Child Death Review files of all 50 U.S. states, media reports, and the CPSC’s National Electronic Injury Surveillance System (NEISS) dataset, which is comprised of emergency room data from approximately 100 representative hospitals across the U.S. [17]. A CPSC investigator conducts a field investigation once a case is identified, a process which culminates in an incident report. Although the CPSC seeks to identify relevant product and manufacturer information if possible, INDPs are also conducted if a specific product, model number, or manufacturer cannot be identified (e.g., an in-ground pool may appear as product-type category “swimming pools, not specified”).

A record of INDP incidents was accessed via a Freedom of Information Act (FOIA) request, which sought all submersion case records of victims 13 years old or younger from 2000–2017. The FOIA was requested winter 2018–19 and the resulting dataset returned summer 2019. Additional FOIA criteria included those cases involving pool-product categories (above ground, portable, wading, unspecified, etc.) as well as categories for spas (i.e., home spas, or hot tubs), swimming pool equipment, inflatable toys, water slides, and other swimming-related incidents.

The resulting record set included several CPSC-specific incident attributes, such as report number, task number, and investigation status. Additionally, the record set included report date, incident city and state, gender and age of patient (in years, or years/months if younger than 2 years old), primary injury category and body part, severity (i.e., fatal or non-fatal), product category, and a narrative, the latter of which was analyzed in this study. The average narrative length was 73 words.

### 2.2. Inclusion Criteria

The initial dataset included 1723 incidents occurring in pool or spa settings. However, 196 cases were removed due to duplicates in the record wherein the same incident was recorded twice due to the implication of more than one aquatic product. Additionally, any narrative that referenced multiple victims was replicated so that one case would represent one child; this resulted in 24 additional cases. Then, all incidents that occurred prior to 2000 but recorded months or years later were excluded (*n* = 3) as well as one that occurred outside the 50 U.S. states (*n* = 1). Seeking to only reflect incidents in structured pool/spa environments, those that occurred in natural water bodies, like rivers and lakes, were excluded (*n* = 8). Finally, apparent errors in the dataset, such as one case referencing an adult fatality and another involving a firework accident, were excluded (*n* = 2). In total, these adjustments resulted in 1537 included cases.

### 2.3. Attribute Development

Attributes were derived from narrative content through an inductive process. An initial independent review was conducted by a member of the research team of the 200 narratives, identifying and defining emergent codes. After this first review, all three research team members met to consider those initial, proposed codes, discuss definitions, and evaluate whether any new or combined codes were relevant. Ultimately, several attributes related to permission (permitted water access), supervision (alone at time of incident, active adult supervision, inactive supervision time), supervision characteristics (supervisor type, supervisor gender, supervisor distraction), and precipitating events (precipitating event) were identified and recorded. Table 1 outlines the attribute definitions and categorial options for each.

Then, the initial researcher returned to the dataset with the refined list of codes, re-evaluated the first 200 narratives, and continued to code narratives in 200-case increments. At the same time and in 200-case batches, a second researcher evaluated the coding of each narrative for accuracy and to confirm consistency in the application of attribute definitions. The three-researcher team met monthly to review cases in which differences in coding were identified. As an indication of improved reliability, the number of cases flagged for potential inconsistency improved monthly. Early batches of 200 cases sometimes had 20+ cases with one or more attributes highlighted as potentially inconsistent; however, the last three batches had 9, 5, and 2 inconsistences flagged, respectively.

### 2.4. Analysis

Descriptive quantitative analysis included counts and frequencies of the narrative codes. SPSS 27.0 was used for statistical analyses, which included chi-square (χ^2^) tests of association and a Mann–Whitney U test for estimated time unsupervised (α set at 0.05). Odds ratios with 95% confidence intervals were also calculated. This study was approved by the Institutional Review Board of Indiana University (2009998119, 29 September 2020). Subject consent was waived due the lack of clearly identifiable or contact information for cases within the INDP database. Narrative quotes were added to illustrate attributes; in these, minor details may have been changed (e.g., gender of victim and/or supervisor, age by a few months) to avoid identifiability of victims.

## 3. Results

The age of victims predominantly represented toddler years: 30.3% (*n* = 465) were 12–23 months, 38.0% (*n* = 583) were 2 years old, and 19.7% (*n* = 303) were 3 years old. Then, 8.2% (*n* = 126) were 4 years old, 3.1% (*n* = 47) were between ages 5–9 years old, and 0.8% (*n* = 13) were 10–12 years old. By gender, 37.3% (*n* = 573) of victims were female, and 62.7% (*n* = 963) were male. Incidents were reported across all 50 U.S. states, excluding North Dakota, with nearly half occurring in the U.S. South (46.4%, *n* = 713). Remaining incidents occurred across the U.S. Midwest (22.4%, *n* = 344), West (17.6%, *n* = 271), Mid-Atlantic (10.8%, *n* = 166), and New England (2.7%, *n* = 42). Only approximately 1 in 10 (10.1%, *n* = 155) involved a hot tub or spa; the remainder are implied to have occurred in a pool (temporary or permanent).

By submersion incident outcome, 1375 (89.5%) resulted in fatal outcomes and 162 (10.5%) resulted in non-fatal outcomes. Among fatal outcome incidents, 29.8% (*n* = 48) of victims were 12–23 months, 35.4% (*n* = 57) were 2 years old, 17.4% (*n* = 28) were 3 years old, 8.1% (*n* = 13) were 4 years old, 5.0% (*n* = 8) were 5–9 years old, and 4.3% (*n* = 7) were 10–12 years old. By non-fatal outcome incidents, 30.3% (*n* = 416) were 12–23 months old, 38.8% (*n* = 526) were 2 years old, 20.0% (*n* = 275) were 3 years old, 8.2% (*n* = 113) were 4 years old, 2.8% (*n* = 39) were 5–9 years old, and 0.4% (*n* = 6) were 10–12 years old.

### 3.1. Permission

Of cases indicating the presence or lack of permission, most incidents reflected a case in which the child did not have permitted water access (86.1%, *n* = 1044; Table 2). This attribute was found to be significantly associated with a fatal outcome: whereas 9.2% (*n* = 100) of fatal incidents indicated permitted water access, 54.8% (*n* = 68) of non-fatal incidents indicated permitted water access χ^2^ (1, N = 1212) = 194.26, *p* < 0.001. Submersion incidents without permitted water access (“No”) were 12 times more likely to have a fatal outcome than those with permitted water access (“Yes”) (odds ratio (OR), 11.98; 95% CI, 7.97–18.06). The example narrative that follows illustrates a case without permitted water access:

“The [2-year-old male] exited the home in the early morning through an exterior door…and went into the backyard where he entered the pool and drowned. The victim’s father was either still in bed or in the bathroom shaving…”

### 3.2. Supervision

Concomitantly, most child victims of submersion incidents were alone at time of incident (80.2%, *n* = 1018; Table 3), contrasting with incidents when another child(ren) was present (13.4%, *n* = 170), an adult was present (5.4%, *n* = 68), or they were in the presence of a larger group or family (1.1%, *n* = 14). Whether a child was alone at time of incident was also significantly associated with the fatality of the submersion incident χ2 (1, N = 1270) = 269.02, *p* < 0.001. Compared to incidents in which an adult(s) was present, those who were alone were 35 times more likely to have a fatal outcome (OR, 34.93; 95% CI, 19.69–61.96). Additionally, incidents occurring with a child(ren) present or “other” present were approximately 10 times more likely to have a fatal outcome (Child OR, 11.46; 95% CI, 5.87–22.38) (Other OR, 9.69; 95% CI, 2.01–46.81). The example narrative that follows illustrates a case reflecting alone at time of incident:

“A 2-year-old boy drowned in a backyard, above-ground pool. He was outside playing with his 3 siblings while the parents were working inside the family’s house. When the oldest sibling realized he wasn’t with them, she went to the backyard, found him floating in the pool, pulled him out, and rushed to the front of the house while calling out for his parents…”

The excerpt above also illustrates a lack of active adult supervision. Of cases exhibiting this variable, nearly all (94.4%, *n* = 1323) indicated the lack of active supervision. Active adult supervision was also significantly associated with the fatality of the submersion incident χ^2^ (1, N = 1402) = 263.81, *p* ≤ 0.001. Submersion incidents without active adult supervision (“No”) were 24 times more likely to have a fatal outcome than those with active adult supervision (“Yes”) (OR, 23.53; 95% CI, 14.20–38.98).

**Figure 1:**
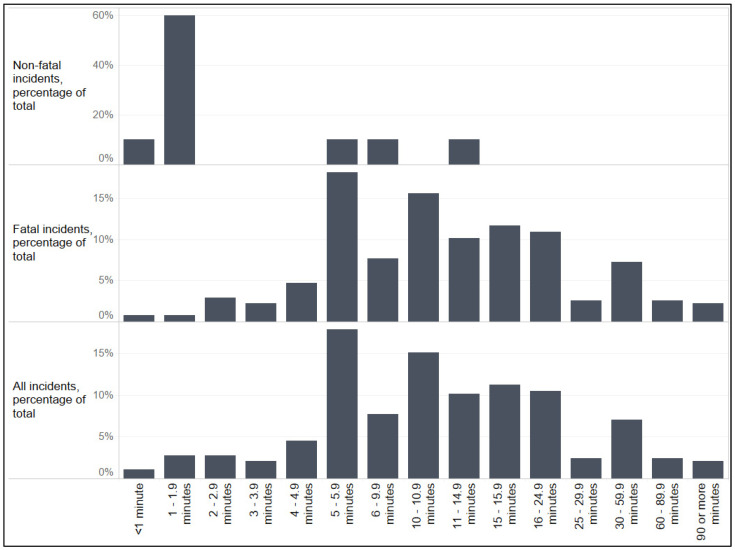
Reported inactive supervision time of U.S. children ages 0–13 submersion incidents investigated by the U.S. Consumer Product Safety Commission, 2000–2017 (*n* = 285).

Many narratives also provided an estimate of inactive supervision time (*n* = 285). Of those narratives including such an estimate, the average time unsupervised was M = 15.6 min (SD = 22.9); however, incidents were reported to have occurred ranging from after less than a minute/immediately (0.1 min) of time transpiring up to nearly four hours (225 min; Figure 1). Notably, inactive supervision time was significantly longer in fatal incidents than non-fatal incidents (U = 326.0, *p* < 0.001).

“A 24-month-old boy was found floating in an above-ground portable pool. The child’s mother stated she had put the child down for a nap, and approximately 5 min later, she noticed the back door was open and her son was not in his room…”

Furthermore, an additional 78 narratives indicated qualitative descriptions of the amount of time unsupervised (e.g., “a short time later” or “a few minutes”); no attempt was made to translate these into a quantifiable period of time, but we note them here and include an example of one such occurrence:

“A three-year female was found face down in a small, inflatable pool after an adult left her unsupervised for a few minutes…”

### 3.3. Supervisor Characteristics

Several characteristics describing the supervisor were also captured in narratives’ text (Table 4). Of those incidents in which the supervisor was specified (*n* = 514), approximately half occurred under the supervisor type of a parent(s) (55.6%, *n* = 286), and approximately one-in-seven occurred under the supervision of a grandparent(s) (14.0%, *n* = 72). Childcare providers, such as babysitters or daycare personnel, were identified as the supervisor in 10.1% (*n* = 52) of known supervisory cases. This variable was not significantly associated with the fatality of the submersion incident χ^2^ (1, N = 514) = 6.35, *p* = 0.500. However, when evaluated on a binary grouping (e.g., parent supervisors vs. non-parent supervisors), the results were significant χ^2^ (1, N = 514) = 4.97, *p* = 0.026, and submersion incidents with a non-parent supervisor were two times more likely to have a fatal outcome than those with parent supervision (OR, 1.97; 95% CI, 1.07–3.64).

The following two examples illustrate the role of two different supervisor type submersion incidents:

“An 18-month-old boy drowned in an above ground swimming pool while unattended. He was last seen by his father in the living room. His father went into another room for 4–5 min. When his father came downstairs, he was not in the living room. His father found him…in the pool in the backyard…”

“A one-year-old girl was found in a residential in-ground jacuzzi…located at the daycare center where they were being cared for…the pool had a wrought iron fence around it, but the gate was not self-closing and not working. The adult care provider left them unattended to use the bathroom.”

By the narrative’s implied supervisor gender (e.g., “mother”), 71% (*n* = 223) of incidents occurred under the supervision of a female supervisor. By outcome, 30% (*n* = 83) of fatal incidents indicated male supervisor gender, and 21% (*n* = 8) of non-fatal incidents indicated male supervisor gender. However, supervisor gender was not found to be significantly associated with the fatality of the submersion incident χ^2^ (1, N = 314) = 1.551, *p* = 0.213. The example that follows illustrates a mother (implied female gender) losing track of a child during the course of typical caregiving activities:

“A five old girl drowned when she climbed into a neighbor’s outdoor, above-ground pool without permission. The girl wandered off while her mother was buying ice cream at an ice cream truck…”

The last supervisor characteristic variable recorded was that of supervisor distraction. In 16% (*n* = 248) of narratives, a distraction was specifically noted, such as a caregiver making lunch, using the bathroom, attending to other household activities/chores, socializing, or relaxing (e.g., watching television). Two examples of such distractions are noted below:

“The two-year-old boy was playing in the backyard while his grandfather worked on his boat. [The grandfather] went inside his shop for 3–5 min, and when he exited, he could not see the victim. He searched for the victim who was found floating in the above-ground, permanent swimming pool.”

“A 2-year-old girl was being watched by her mother…the mother was in the kitchen preparing lunch while the child was in the living room. A few minutes later, the mother noticed the victim was not in the living room and went to look for her. The victim was found in an above-ground swimming pool in the backyard…”

“This incident involved a 17-month-old boy who drowned in his family’s backyard spa. The victim had played in the yard much of the day with his mother. He had many toys in the yard and the spa. They went inside when it got dark. The mother used the bathroom for 5 min and the boy went outside, unseen through a pair of unlocked French doors...The mother found the boy unresponsive.”

### 3.4. Precipitating Events

Finally, precipitating events may contextualize the efficacy of supervision or permission characteristics (Table 5). There were 315 incidents that explicitly identified a precipitating event. Among these, nearly half described that the child was engaged in outdoor play at the time of the incident (45.7%, *n* = 144). An example of an outdoor play precipitating event follows.

“A 15-month-old female drowned in a portable, backyard swimming pool while supervised. The victim was last seen playing outside in the backyard...the pool ladder was in place to access the pool. She was subsequently discovered by her brother in the pool, unresponsive…”

Approximately one-third of submersion incidents with an identified precipitating event were characterized as having occurred during social gathering or party, such as a birthday party (35.6%, *n* = 112); below is an illustrative example of one such case:

“A 14-month-old male victim drowned in the backyard of his grandparents’ home during a family barbeque. The victim was inside the house, but the grandmother lost track of him for less than 5 min. The victim was found face down in a small, inflatable, plastic pool…The victim wondered outside through an open door and drowned in 7 inches of water.”

Finally, nearly one-in-six (15.2%, *n* = 48) were situations in which the child had previously engaged in permitted water play, usually that same day, but later returned to the pool/spa setting without supervision or permission, resulting in a submersion incident. The narrative below illustrates an example of previous water play as a precipitating event:

“The 18-month-old male victim, his mother, and cousins were in the backyard playing in a 10-foot inflatable pool. The mother [then] put the victim and his six-year-old cousin in their room to play with some toys. She went to the kitchen to check on dinner and make a phone call. About 10 min later, she went to check on the children and found her daughter had disappeared. She found the victim in the pool, floating face down.”

## 4. Discussion

In this study, we adopted an epidemiological lens of available narrative data to understand how factors related to permission, supervision, and precipitating events contribute to submersion incidents. Seeking to contribute to the body of work among researchers, activists, and advocates that aims to reduce pediatric drownings, we evaluated 1537 youth submersion incident reports from 2000–2017. These attributes relating to permission, supervision, and precipitating events elucidate noteworthy findings.

Our results indicated that 86% of evaluated incidents occurred without permitted water access and that these incidents were 12 times more likely to result in a fatal outcome than those with permitted water access. Among fatal outcomes, 90.1% occurred without permitted water access, in contrast to 45.2% of non-fatal incidents. Findings associated with alone at time of incident were comparable; approximately four out of five incidents occurred with a child was alone, although this differed based on the fatality of the outcome (40.9% of non-fatal outcomes vs. 83.9% of fatal outcomes). These incidents in which a child was alone were 35 times more likely to have a fatal outcome than those in which an adult(s) was present. A substantial portion of events also occurred without active adult supervision (94.4% overall, 61.7% of nonfatal incidents, and 96.6% of fatal incidents), and those without active adult supervision were 24 times more likely to result in a fatal outcome than those with active adult supervision. These findings associating the lack of active adult supervision with submersion incidents generally align with other scholarship, lending credence to the validity of our results [12,13]. Yet, these findings also contextualize the burden put on caretakers regarding the need for hypervigilant supervision of (particularly young and newly mobile/ambulatory) children [18], especially in light of other household pressures [19]. Indeed, in a position paper by the U.S. National Drowning Prevention Alliance’s Education Committee, they stated that during non-water activities, they emphasize that child supervisors should “ALWAYS know where children are,” representing a herculean task [11].

The predominance of parents as the most frequent supervisor type (55.6%) during submersion incidents aligns with their general role as primary caregivers as well as extant literature on childhood drownings [14,20]. Indeed, Shenoi et al.’s [20]’s examination of 260 youth swimming pool submersions in Harris County, Texas, from 2003–2007 found that parents were the supervising individual 60% of the time. Our findings also found that when grouped by parent vs. non-parent supervisors, those incidents without parent supervision were two times more likely to result in fatality. Because of this, the role and occurrence of grandparents (14.0%), childcare providers (10.1%), and other adults (9.5%) in our data are also noteworthy. We understand that this study may be the first to identify a more specific supervisor type. Grandparents, in particular, often act as both “backup parent” and relative; approximately 50% of young children spend time with a grandparent weekly [21]. The frequency of submersion under the supervision of a childcare provider (e.g., daycare provider, babysitter) is also particularly noteworthy and may warrant targeted communication efforts to both providers and parents indicating the risk of pools and spas, particularly when care is provided outside of the home, where the pool/spa danger is less apparent.

Supervisor gender has also not previously been evaluated systematically, although others [14] have coded via a system implying parent gender but not the gender of other supervisors (e.g., mother, father, parent supervisor—unclear, grandparent, sibling, extended family member, etc.). In that work, among incidents in which the parent/supervisor gender is known, 67.3% occurred under the supervision of the mother [14]. Similarly, our results indicated that 71.0% of known supervisor gender incidents were female, although this differed only slightly (and non-significantly) by nonfatal (79.5%) and fatal (69.8%) outcomes. Importantly, however, rather than implying that female rescuers are more likely to provide inadequate supervision, we suspect that this aligns with data indicating women spend more time supervising children [22,23]. To that end, in 16.1% of incidents, a specific supervisor distraction was noted, such as the narrative indicating a mother using the bathroom or another preparing lunch. Indeed, a cursory qualitative review of such cases indicated that many distractions were associated with a task necessary to household functioning. As such, calls for layers of barriers that would prevent water access even during a temporary lack in supervision are supported here [11].

The final precipitating events attribute sought to understand greater social and behavioral context during or prior to the submersion incidents. Among those incidents indicating a precipitating event, the frequency of outdoor play (45.7%) as a precipitating event suggests that proximity to the outdoor pool or spa may be key, as children who are already outdoors (perhaps beyond one or more barriers, such as a locked house door) may enter an accessible pool/spa without permission or knowledge of a supervisor. In contrast, the social gatherings pattern (35.6%)—such as birthday parties or cook-outs—suggest ineffective supervision systems wherein the presence of multiple individuals may provide a false sense of security, as no one individual may be specifically charged with supervising the child(ren) or water body. In Peden and Franklin [14]’s investigation of supervision distractions at aquatic locations, they found that among those attributed to “talking/socializing”, nearly 60% occurred at a swimming pool, further substantiating the link between pool/spa settings, distractions attributed to social activities, and child submersion incidents. Finally, shifting attention to behavioral motivations of the child, the frequency of previous water play suggests a child gained access to and re-entered the pool/spa where they had previously been playing safely, most likely under supervision. This finding suggests a conditional hazard: a pool or waterbody is often a permitted space where fun play occurs while also posing an imminent and fatal danger to young children when access is not permitted and/or unsupervised. The frequency of previous water play (15.2%) in child submersion incidents aligns with Morrongiello et al.’s [24]’s proposition that conditions “that allow for inconsistency in child risk behavior, therefore, may be particularly likely to elevate young children’s risk of injury” (p. 259). Still, while these patterns in precipitating events are noteworthy, approximately 80% of all cases did not indicate a precipitating event, whether due to underreporting (i.e., based on an investigator’s subjective decision over what to include in the narrative) or the lack of such an event occurring. Future efforts seeking to refine or improve drowning-related data collection should consider the inclusion and standardization of these relevant circumstantial characteristics.

## 5. Limitations

Convenience sampling and the need for parent/caregiver consent to the field investigation are two limitations in this approach. Additionally, non-fatal events are likely underreported given that more minor events may not trigger an INDP investigation or even hospitalization. Ideally, a more complete dataset would have a more representative sample of nonfatal and fatal incidents. This is one challenge associated with using a pre-existing dataset of this kind. Still, an endeavor to collect this type and quantity of data independently as researchers would likely have been dramatically more resource-intensive, if not insurmountable. Furthermore, the accuracy of some attributes, such as inactive supervision time, represent recall estimates; and as mentioned previously, other attributes, such as precipitating events, may be underreported. In many cases, one or more attributes were “unspecified”; however, this indicates the lack of reporting in field investigators’ written narratives and not necessarily the lack of the phenomenon. Still, despite the sampling strategy reflected in the INDP’s curation, the alignment with our results on this larger sample and extant literature outlined herein point to generalizability of our findings.

## 6. Conclusions

To our knowledge, this study is the first large-scale effort to employ investigator narrative accounts to examine the role of permission, supervision, and precipitation events in youth submersion incidents. Despite some limitations associated with this approach, our use of the CPSC’s pre-existing INDP dataset allowed for the detailed review of more than 1500 submersion incidents across the U.S. Our results suggest that future educational and outreach efforts seeking to prevent childhood drownings should not only target parents but other individuals that supervise children (e.g., grandparents, child caregivers) in communicating the risk of proximate pools and spas. These efforts should reiterate key findings, such as (1) most submersion incidents occur when the child does not have permission to enter the pool/spa and when an adult is not actively supervising the child, (2) dangerous submersion incidents can occur in just a few minutes, and (3) submersion incidents frequently occur when a supervisor is distracted—such as during an outdoor social gathering—or when a child, unbeknownst to a supervisor, is playing outdoors or returning to a pool/spa where they had previously been permitted access. Given these factors, as well as the very young age of most submersion victims in this sample, the cruciality of adults’ diligent supervision and understanding of factors like precipitating events cannot be understated. Additionally, the young age at which many incidents occurred indicates that deployment of multiple strategies regarding accessibility and supervision are warranted, as young children may physically be able to access a pool or spa but not cognitively understand the danger posed by non-permitted access. This “multiple strategies” tactic aligns with expert calls to engage multiple layers of protection, including passive measures, like barriers and alarms, and active measures, like diligent adult supervision [25]. In cases where permission is lacking or supervision lapses, an effective barrier may be the differentiating factor in whether there is a tragic outcome. Finally, we hope that researchers and professionals working in water safety would understand that the findings outlined here may be illustrative of characteristics related to permission, supervision, and precipitating events in other drowning locations, such as natural waterbodies or public pool spaces or even other childhood-injury prevention areas. Ultimately, through this research, we hope that these findings aide in the design of educational campaigns and prevention efforts to reduce youth submersion incidents.

## Figures and Tables

**Table 1:** Attribute Descriptions.

Attribute	Definition	Categories
**Permission**		
Permitted Water Access	Whether child was known/permitted to be at the water body of the incident, i.e., in permitted pool play or swimming. Note: If the mechanism of access was mentioned (e.g., unlocked gate), this implies unpermitted access	Yes No Unspecified
**Supervision**		
Alone at Time of Incident	Whether another individual was present at time of incident; “alone” may be implied if child (a) “was found” and (b) there was an explicit mention of how child accessed the pool/spa on their own. Child(ren) present was generally based on an explicit description of a child’s age (e.g., “with his 12-year-old brother”) or in fewer cases, an implied description (e.g., “a 4 year-old-boy and his two friends”)	Alone No—adult(s) present No—child(ren) present No—other, e.g., group or family Unspecified
Active Adult Supervision	Whether victim was actively being supervised by an adult at the time of the incident (e.g., in the same room, outdoors at the pool with child) (note: terms such as “was found” implies lack of active supervision)	Yes No Unspecified
Inactive Supervision Time	Approximate time estimated that the supervisor did not have “eyes” on the child, in minutes (note: if range is provided, average is calculated)	Continuous, in minutes
**Supervisor Characteristics**		
Supervisor Type	Individual implied to be in charge of victim’s safety, whether or not present at time of incident, (e.g., “was watching,” “left with older sister”)	Parent(s) Grandparent(s) Childcare provider Unspecified adult(s) Unspecified child(ren) Relative Sibling Other Unspecified
Supervisor Gender	Implied gender of supervisor (e.g., mom, aunt = “female”)	Male Female Unspecified
Supervisor Distraction	Whether a specific distraction was noted for the supervisor	Yes Unspecified
**Precipitating Events**		
Precipitating Event	Whether victim was engaged in a precipitating event or related activity during or prior to the submersion incident	Social gathering Outdoor play Previous water play Other Unspecified

**Table 2:** Permission.

Description	All Cases		Non-Fatal		Fatal		*p*-Value
	*n*	%	*n*	%	*n*	%	
**Permitted Water Access**							
Yes	168	13.9%	68	54.8%	100	9.2%	<0.001
No	1044	86.1%	56	45.2%	988	90.8%	
**Total**	**1212**	**100.0%**	**124**	**100.0%**	**1088**	**100.0%**	
Unknown	325		38	-	287	-	

**Table 3:** Supervision.

Description	All Cases		Non-Fatal		Fatal		*p*-Value
	*n*	%	*n*	%	*n*	%	
**Alone at Time of Incident**							
Yes	1018	80.2%	45	40.9%	973	83.9%	<0.001
No—child(ren) present	170	13.4%	21	19.1%	149	12.8%	
No—adult(s) present	68	5.4%	42	38.2%	26	2.2%	
No—other e.g., group, family	14	1.1%	2	1.8%	12	1.0%	
**Total**	**1270**	**100.0%**	**110**	**100.0%**	**1160**	**100.0%**	
Unknown	267	-	52	-	215	-	
**Active Adult Supervision**							
Yes	79	5.6%	46	38.3%	46	3.4%	<0.001
No	1323	94.4%	74	61.7%	1323	96.6%	
**Total**	**1402**	**100.0%**	**120**	**100.0%**	**1369**	**100.0%**	
Unspecified	135	-	42	-	93	-	
**Inactive Supervision Time (minutes)**							
*n*	285		10		275		<0.001
Mean (SD)	15.6 (22.9)		3.2 (3.8)		16.1 (23.2)		
Minimum, median, maximum	0.1, 10, 225		0.1, 1.3, 12.5		0.5, 10, 225		

**Table 4:** Supervisor Characteristics.

Description	All Cases		Non-Fatal		Fatal		*p*-Value
	*n*	%	*n*	%	*n*	%	
**Supervisor Type**							
Parent(s)	286	55.6%	37	69.8%	249	54.0%	0.500 ^1^
Grandparent(s)	72	14.0%	4	7.5%	68	14.8%	
Childcare provider	52	10.1%	4	7.5%	48	10.4%	
Unspecified adult(s)	49	9.5%	5	9.4%	44	9.5%	
Unspecified child(ren)	21	4.1%	1	1.9%	20	4.3%	
Relative	19	3.7%	1	1.9%	18	3.9%	
Sibling	7	1.4%	0	0.0%	7	1.5%	
Other	8	1.6%	1	1.9%	7	1.5%	
**Total**	**514**	**100.0%**	**53**	**100.0%**	**461**	**1**	
Unspecified	1023	-	109	-	914	-	
Supervisor Gender							
Female	223	71.0%	31	79.5%	192	69.8%	0.213
Male	91	29.0%	8	20.5%	83	30.2%	
**Total**	**314**	**100.0%**	**39**	**100.0%**	**275**	**100.0%**	
Unspecified	1223	-	123	-	1100	-	
Supervisor Distraction							
Yes	248	16.1%	21	13.0%	227	16.5%	-
Unspecified	1289	83.9%	141	87.0%	1148	83.5%	
**Total**	**1537**	**100.0%**	**162**	**100.0%**	**1375**	**100.0%**	

^1^ Statistical significance identified when evaluated based on binary grouping (parent vs. non-parent supervision, *p* = 0.026).

**Table 5:** Precipitating Events.

Description	All Cases		Non-Fatal		Fatal	
	*n*	%	*n*	%	*n*	%
Social gathering	112	35.6%	11	34.4%	101	35.7%
Outdoor play	144	45.7%	16	50.0%	128	45.2%
Previous water play	48	15.2%	3	9.4%	45	15.9%
Other	11	3.5%	2	6.3%	9	3.2%
**Total**	**315**	**100%**	**32**	**100%**	**283**	**100%**
Unspecified	1236	-	131	-	1105	-

Note: Total exceeds number of narratives, as multiple devices were recorded in some cases.

## Data Availability

Data was retrieved via a Freedom of Information Act submitted to the CPSC. As the dataset could potentially be used to identify child submersion victims and/or their caregivers, the underlying data will not be made available.
